# Phylogenetic analysis of viruses in Tuscan *Vitis vinifera sylvestris* (Gmeli) Hegi

**DOI:** 10.1371/journal.pone.0200875

**Published:** 2018-07-18

**Authors:** Erika Sabella, Roberto Pierro, Andrea Luvisi, Alessandra Panattoni, Claudio D’Onofrio, Giancarlo Scalabrelli, Eliana Nutricati, Alessio Aprile, Luigi De Bellis, Alberto Materazzi

**Affiliations:** 1 Department of Biological and Environmental Sciences and Technologies, University of Salento, via Prov.le Monteroni, Lecce, Italy; 2 Department of Agriculture, Food and Environment, University of Pisa, Via del Borghetto, Pisa, Italy; Universidade de Lisboa Instituto Superior de Agronomia, PORTUGAL

## Abstract

The health status of the native grapevine *Vitis vinifera* subsp. *sylvestris* (Gmeli) Hegi in natural areas in Europe has received little attention. A survey was carried out on wild grapevines in Tuscany (Italy), where isolates of the *Grapevine rupestris stem pitting virus* (GRSPaV), *Grapevine leafroll-associated virus 1 and 3* (GLRaV-1 and GLRaV-3) and *Grapevine virus A* (GVA) were detected. The complete coat protein (CP) region of these isolates was sequenced to investigate the relationship of the viral variants from Tuscan wild grapevines with isolates from different geographical origins. According to the phylogenetic analyses, GLRaV-1 and GLRaV-3 isolates from Tuscan wild grapevines clustered with isolates from cultivated grapevines with nucleotide sequence identities ranging from 66% to 87% and from 72.5% to 99% respectively, without any correlation between the distribution and geographical origin. Conversely, GRSPaV and GVA isolates clustered together with other Italian isolates from *V*. *vinifera* with nucleotide sequence identities ranging from 71.14% to 96.12% and from 73.5% to 92%, respectively. Our analysis of the whole amino acid sequences revealed a high conservation level for the studied proteins explained by a selective pressure on this genomic region, probably due to functional constraints imposed on CP, such as specific interactions with cellular receptors in the insect vectors necessary for successful transmission. In addition, analyses of genetic recombination suggest no significant point mutations that might play a significant role in genetic diversification. The d*N*/d*S* ratio also estimated a low number of non-silent mutations, highlighting the purifying selective pressure. The widespread distribution of the Rugose wood complex (GRSPaV and GVA associated disease) in comparison with the Grapevine Leafroll associated viruses (GLRaV-1 and -3) could explain the major geographical correlation found for the viral variants detected in Tuscany.

## Introduction

Grapevine (*Vitis* spp.) domestication goes back five thousand years and today, this fruit crop has spread worldwide and has a high socioeconomic importance. A significant limiting factor to grapevine production is its susceptibility to many agents causing diseases, some of which reduce the health and fruit quality of the plant with a resulting crop loss. Among these agents, many viral species and strains have been associated with economically important constraints to grape production. These pathogens can be graft- and vector-transmitted (mealybugs, aphids, thrips, leafhopper, etc.). Thus not only do the frequent exchanges of propagative material among countries contribute to the spread of these pathogens, but unmonitored wild plants that may act as a reservoir of pathogens represents a risk for the disease in grapevines [1].

The perennial wild grape *Vitis vinifera* L. subsp. *sylvestris* (Gmeli) Hegi is assumed to be the progenitor of cultivated grapevines and a few populations still exist, despite it being an endangered taxon, above all as a consequence of habitat degradation due to human activities. These populations can be a natural source of pathogens including viruses because their perennial life cycle further accelerates the mixing and introduction of several viral agents into a single plant [1].

The health status of wild grapevine in Europe areas has not received sufficient attention [1]. Surveys on grapevine virus infections are available from Portugal [2,3], Austria [4] and Italy [1], and a few accessions have been assayed in France [5]. Recently, surveys aimed at identifying viruses present in wild and native *Vitis* germplasm were carried out to shed light on the presence of new viruses and to analyze the possible impact of these viruses on cultivated *Vitis* spp. [6].

Not all viruses infecting *Vitis vinifera* cultivars cause economically important diseases.

European Commission directive 2005/43/EC considers infectious degeneration [*Grapevine fanleaf nepovirus* (GFLV), *Arabis Mosaic virus* (ArMV)], leafroll [*Grapevine leafroll associated-virus 1* (GLRaV-1), *Grapevine leafroll associated-virus 3* (GLRaV-3)], fleck [*Grapevine fleck virus* (GFkV), only for rootstocks] and the related viruses ascertained as causal agents, as harmful diseases whose absence in nursery stocks must be confirmed through official inspections. Italy adopted directive 2005/43-EC but with other added viruses for grapevine certification, such as *Grapevine virus A* (GVA) (Italian Ministry decree DM 07/07/2006) and *Grapevine virus B* (GVB) (Italian Ministry decree DM 24/06/2008), which are associated with rugose wood, as well as *Grapevine leafroll-associated virus 2* (GLRaV-2) (Italian Ministry decree DM 24/06/2008). None of these viruses have been reported in wild grapevine *Vitis vinifera* subsp. *sylvestris* in European surveys, while viruses included in EU and Italian regulations are distributed worldwide in cultivated *V*. *vinifera* [7].

*Grapevine rupestris stem pitting virus* (GRSPaV) also has a worldwide distribution although it is not included in certification [7,8]. According to previous studies, GRSPaV also seems to be the most widespread in wild grapevines (12–31%) [2,1]. In Italy, only Sicily (southern Italy) has been extensively monitored for wild grapevines, with GRSPaV and GFLV as the sole viruses found. Although these grapevine disease causal agents are also present in Italian vineyards, where GRSPaV was found to be the most widespread virus, GFLV infections are quite limited compared to leafroll, fleck and rugose wood [9,10].

GRSPaV belongs to the family *Betaflexiviridae*, subfamily *Quinvirinae* genus *Foveavirus* [11] and is associated with distinct diseases: Rupestris stem pitting (RSP) [12] and vein necrosis [13].GRSPaV is frequently found in association with other viruses [14], otherwise, the virus is usually detected in *V*. *vinifera* L. cultivars in a dormant state, generally, with mild or no specific symptoms [15]. The virus was also shown to have beneficial effects such as increase in transcripts involved in photosynthesis and CO_2_ fixation, [16].

GLRaV-1 and GLRaV- 3 belong to the family *Closteroviridae*, genus *Ampelovirus* and are among the most widespread grapevine leafroll associated-viruses (GLRaVs). GLRaVs cause alterations in the plant physiological processes resulting in a reduction in yield and crop quality. The viruses colonize the grapevine phloem tissue and interfere with the flow of nutrients to shoots, leaves, and fruit pedicels. This interruption in vascular tissue reduces vigor and prevents the accumulation of sugars and other metabolites. GLRaVs are transmitted by phloem sap-sucking insects [1]. GVA and GVB belong to the family *Betaflexiviridae*, subfamily *Trivirinae* genus *Vitivirus* and cause diseases characterised by grapevine graft incompatibility and wood alterations (i.e. rugose wood) [6]. In the field, these viruses are often associated with GLRaV infection and are transmitted by phloem sap-sucking insects [6].

In this paper, we report the health status of wild grapevines collected in Tuscany. Phylogenetic analysis of the viruses found provides a partial characterization of the local viral isolates. In addition, this work emphasizes the infection risk derived from wild grapevines as an inoculation source, and provides the basis for future epidemiological studies.

## Materials and methods

### Plant material

Forty-four wild grapevines were selected in 2012 in ten natural areas of Tuscany (above all in areas near Grosseto and Siena), which were identified as *V*. *vinifera* subsp. *sylvestris* [17,18]. Plants were checked for symptoms during both the summer and winter surveys for three years. In winter, dormant woody canes were collected for both molecular and serological tests.

### Virus assay

Plant health was evaluated via quantitative real-time PCR (qPCR) and an ELISA test. To account for the possible uneven distribution of viruses within a vine, samples from at least six different shoots were randomly collected and combined.

Total RNA was extracted from cambial scrapings of lignified cuttings (2g) using RNeasy Plant Mini Kit (Qiagen, Netherlands) protocol, modified according to MacKenzie et al. [19]. Tissues (2 gr) were ground using a Tissue lyser (Qiagen) adding 5 ml of grinding buffer (4.0 M guanidine isothiocyanate, 0.2 M sodium acetate pH 5.0, 25 mM EDTA, 2.5% PVP-40 and 2.0% sodium bisulfite added just before use). The homogenate (1 ml) was transferred into a 1.5 ml tube and 100 μl of 20% sarkosyl were added. After 2 min. centrifugation, 600 μl were transferred to a QIAshredder spin column (Qiagen) placed in a 2 ml collection tube. The RNA extraction steps were in line with the manufacturer’s protocol.

The extracted RNA was then retro-transcribed into cDNA using the iScript cDNA synthesis kit (Biorad, USA). Primers and probes for GLRaV-1, GLRaV-2, GLRaV-3 [20], GRSPaV, GVA [21], GFLV, GFkV [22] and ArMV [23] detection were used. For each sample, 2 μl of cDNA were amplified in a total volume of 20 μl containing 1x SsoFasto probe Master Mix (Biorad) and 0.4 μM of each primer and probes. Reactions were performed in a CFX96 real-time thermocycler (Biorad). Samples were positive when the threshold cycle (Ct) values were lower than 34. Negative template controls and blank negatives were used to assess background signal.

Immunoassays were performed following Faggioli et al. [24]. Commercial diagnosis kits for GLRaV-1, -2, -3, GVA, GFkV, GFLV (Agritest, Bari, Italy: catalog number S01B), ArMV (Agritest: catalog number K02B) and GRSPaV (Creative Diagnostic, New York, USA, DEIAPV164) were used. Cambial scrapings of lignified cuttings (2 g) were carried out and mechanically ground (Tissue Lyzer with 10 ml-grinding jar, Qiagen, Venlo, Netherlands) with the extraction buffer supplied by the ELISA kit manufacturing companies. The ELISA tests were conducted following company’s instructions by using commercial polyclonal antibodies as well as negative and positive controls [25]. Samples were positive when the OD_405_ values were at least two times higher than the OD_405_ value of the negative control.

### Virus sequencing and analysis

The amplification of the viral coat protein (CP) gene for GLRaV-1, GLRaV-3, GVA and GRSPaV was performed using the following primers: GLRaV-1-CP/F (5’-CGCGCTTGCAGAGTTTAAGTGGTT-3’) and GLRaV-1-CP/R (5’-TCCGTGCTGCATTGCAACTTTCTC-3’) for GLRaV-1 [26]; LR3-8504V (5’-ATGGCATTTGAACTGAAATT-3’) and LR3-9445C (5’-CTACTTCTTTTGCAATAGTT-3’) for GLRaV-3 [27]; GVA-CPF6356 (5′-GATACYCTAGTTATGCCAGA-3′) and GVA-CPR7096 (5′-GCACCACACTTACACACATTC-3′) covering the full-length GVA coat protein (CP) gene [28]; RSP52 (5’-TGAAGGCTTTAGGGGTTAG-3’) and RSP53 (5’-CTTAACCCAGCCTTGAAAT-3’) for GRSPaV [29]. The CP specific DNA fragments were gel-purified and cloned in a TOPO TA Cloning® Kit for Sequencing (Invitrogen, Carlsbad, USA). The ligation products were transformed into DH5α chemically competent *Escherichia coli* cells according to the manufacturer’s instructions (Invitrogen) and recombinant clones were screened via PCR. Plasmids from selected clones were isolated using QIAprep Spin Miniprep Kit (QIAGEN, Hilden, Germany) and sequenced (Eurofins Genomics, Ebersberg, Germany). Raw sequences were manually edited with GENtle software version 1.9.4 with each base position sequenced at least twice.

The BLAST function in the database of the National Centre for Biotechnology Information was used for sequence comparison. Nucleotide sequences of GLRaV-1, GLRaV-3, GVA and GRSPaV isolates were compared to sequences of isolates (specific reference isolates on which lineage grouping virus variants are based) from different geographical origins retrieved from GenBank (S1–S4 Tables). Multiple Sequence Allignments of the nucleotide sequences were carried out by using ClustalW function on the website of the Kyoto University Bioinformatics Center (https://www.genome.jp/tools-bin/clustalw) employing default parameters. The obtained text files have been converted in the Mega format to be used as data input files for the phylogenetic analysis.

Phylogenetic analyses were carried out using the neighbour-joining (NJ) option (with 1000 bootstrap replicates) of MEGA software version 7 [30]. In addition, d*N*/d*S* ratio was estimated using the codon-based Z-selection test using the Muse-Gaut model with Mega7 (bootstrap 1000 replicates), in order to calculate the probability of rejecting the null hypothesis of strict-neutrality (d*N* = d*S*) in favor of the positive selection hypothesis (d*N* > d*S*). d*S* and d*N* are the numbers of synonymous and non-synonymous substitutions per site, respectively. An overall *dN/dS* ratio > 1.0 and *p* value < 0.05 indicate positive selection, while a ratio = 1 and *p* value < 1.0 indicate neutral or purifying selection process [31,32].

### Recombination analyses

The nucleotide sequences of all the isolates were aligned with sequences of isolates from different geographical origins retrieved from GenBank (S1–S4 Tables) and checked for incongruent relationships to detect putative recombinant isolates using the Recombination Detection Program v.3.34 (RDP4). Given a set of aligned nucleotide sequences, RDP4 provides extensive information on which sequences in the processed dataset carry evidence of the same recombination event, the expected positions of recombination breakpoints, and the identities of sequences that are most closely related to the parental sequences. In addition to the original RDP method, these procedures involve BOOTSCAN, MAXCHI, CHIMAERA, 3SEQ, GENECONV, LARD, and SISCAN. Following the detection of a ‘recombination signal’ with these procedures, the program resolves approximate breakpoint positions using a hidden Markov model, BURT, and then identifies the recombinant sequence using the PHYLPRO, VISRD, and EEEP methods [33]. Default RDP4 settings were used.

## Results and discussion

### Virus assay

No putative disease symptoms were observed either during summer or winter field inspections. These observations are in accordance with data reported in other studies related to the *V*.*v*. *sylvestris* health status. Pacifico et al. [1] described no observed symptoms in Sicilian native grapevines. Nolasco et al. [3] found an asymptomatic GRSPaV infection in Portuguese wild grapevines. In Garfi et al. [34], several surveys were carried out to analyse the population of *V*.*v*. *sylvestris* sampled in different areas of Sicily, and almost all grape plants looked healthy without any evidence of grapevine pathogens. The absence of the typical symptoms in infected wild grapevines seems to suggest a putative tolerance.

No differences between ELISA (S1 File) and qPCR (S2 File) were observed since serological and molecular tests confirmed the same results on the analysed samples. Usually, the same results were obtained by qPCR and ELISA tests but in some cases, viruses could be detected by only one of the two techniques. For example, according to the phylogenetic analyses of the coat protein gene, GLRaV-3 isolates are classified into six groups (I—VI) but recently new groups have been identified (groups VII and VIII); these variant groups include isolates characterized by a greater genetic diversity than those in other groups and these diversities makes them undetectable; according to Maree et al. [35] the isolate GH24 (KM058745, group VII) has been detected in the analysed sample only through ELISA while all effort to amplify the sample via qPCR failed. Thus, beside lower sensitivity, ELISA test should limit the risk of missing some virus isolates.

**Fig 1:**
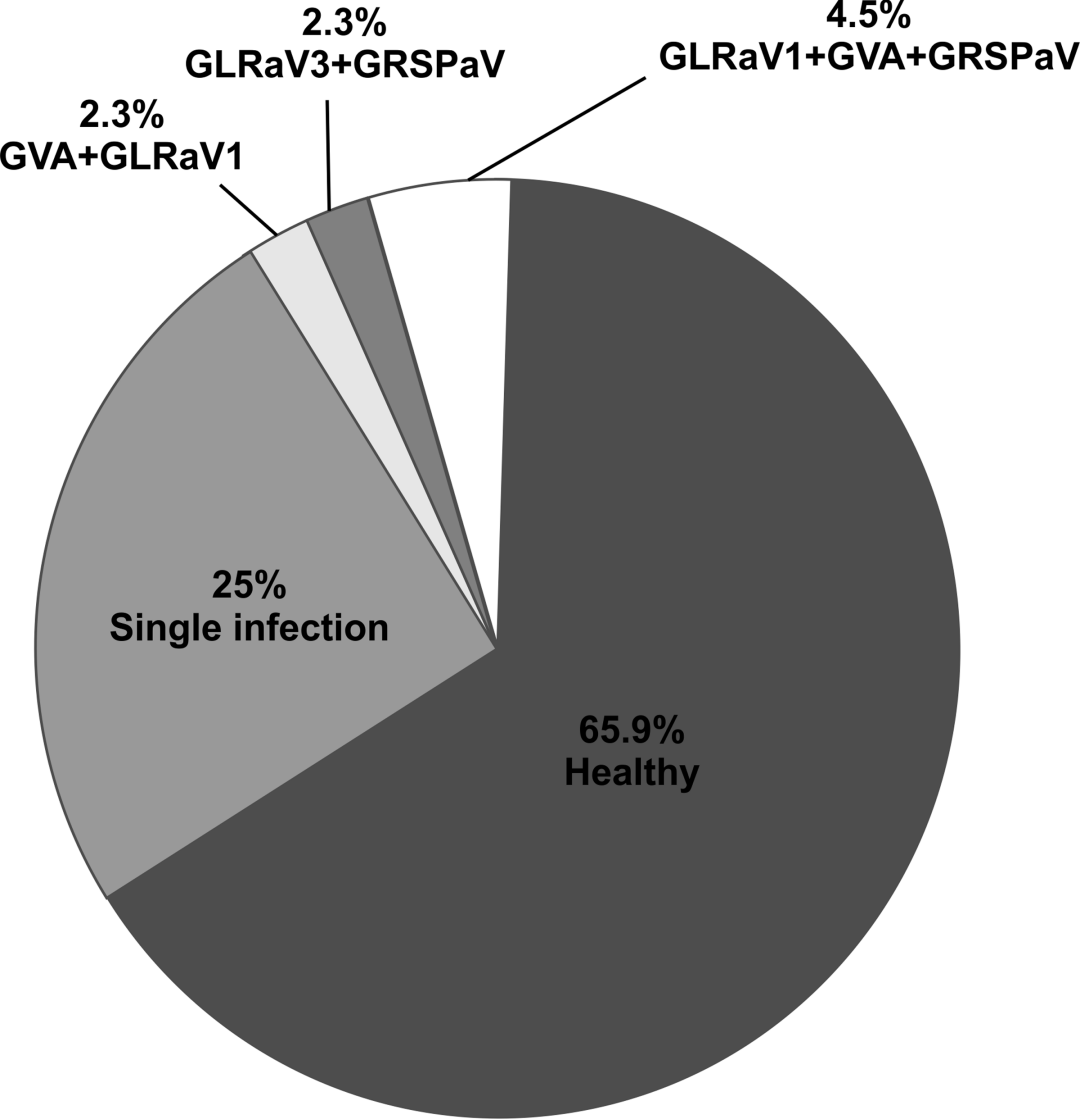
Distribution of single and mixed infections in *Vitis vinifera* subsp. *sylvestris* (Gmeli) Hegi in Tuscany. Detected infections with single and mixed infection (expressed as % of virus out of total number of analysed plants).

Distributions of viruses are reported in Fig 1. Among the diseases, rugose wood was the most prevalent and affected more than 38% of plants, followed by leafroll (14%). The non-regulated GRSPaV was the most prevalent virus with a presence in 30% of plants, while GLRaV-1 was the most frequent among EU-regulated viruses followed by GLRaV-3. The Italian-regulated GVA was found in almost 7% of wild grapevines. No infections with the viruses GLRaV-2, GFLV, ArMV and GFkV have been detected (Fig 1). About one-third of infected samples were mixed infections with a combination of leafroll-associated viruses, GVA and GRSPaV being the most common.

### Virus sequencing and analysis

#### GRSPaV sequences

A considerable molecular variability of GRSPaV resulted in the recognition of distinct phylogenetic groups [3,36,37]. In Terlizzi et al. [36] the analysed sequences shared 77.1–98.6% of nucleotide sequence identity and the phylogenetic analysis generated a tree that revealed four major clusters: groups I, II, III and IV (these lineages correspond, respectively, to groups 2b, 2a, 3 and 1 reported by Nolasco et al. [3]). In this system, the four group of sequence variants were designed by Arabic numerals; in the other system used in literature [37], these groups were identified by the names of the reference isolates for which the entire genomes were sequenced, respectively: GRSPaV-1, GRSPaV-SG1, GRSPaV-BS and GRSPaV-SY; Meng and Rowhani [37] suggested the existence of another group (GRSPaV-XX) but this needs to be verified by sequencing the genomes of the group’s isolates [37]. Groups GRSPaV-1 and GRSPaV-SG1 shared a high nucleotide sequence identity (87.7–93.6%) compared with other groups, suggesting that they had merged into one cluster. The majority of isolates used in the phylogenetic analyses were collected from *V*. *vinifera* and *V*. *labruscana*. Among the isolates from *V*.*v*. *sylvestris*, three were from Portugal and clustered in group GRSPaV-SY and one was from Italy which clustered in group GRSPaV-SG1.

**Fig 2:**
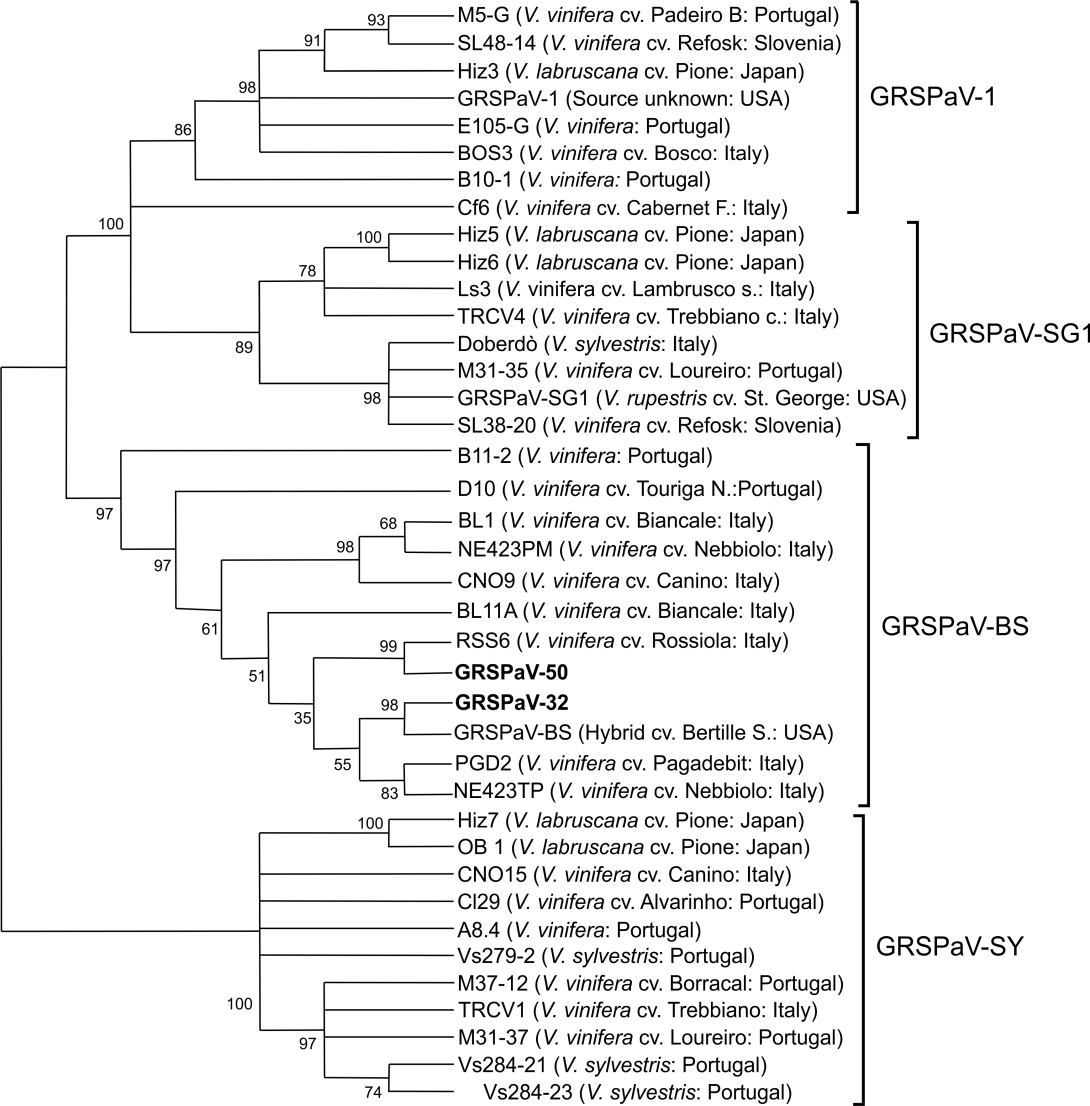
Phylogenetic tree showing the distribution of Tuscan *Grapevine rupestris stem pitting-associated virus* (GRSPaV) coat protein nucleotide sequences. Neighbour-joining tree shows the distribution of Tuscan GRSPaV coat protein nucleotide sequences compared to isolates from the GenBank. Geographical origin is given for each isolate (in brackets). Bootstrap values are from 1000 replicates.

According to our phylogenetic analyses, the two Tuscan GRSPaV isolates (all the isolates identified from the 13 infected plants were sequenced and only the two representative isolates were included in the tree) (S3 File) from native *V*. *v*. *sylvestris* clustered in group GRSPaV-BS (Fig 2), sharing a nucleotide sequence identity of 89% with each other and in a range from 71.14% and 96.12% compared with the other nucleotide sequences from Genbank. The overall ratio between the non-synonymous to synonymous mutations (d*N*/d*S*) for the nucleotide sequences analyzed was < 1 with low number of non-silent mutations, suggesting a purifying selection process.

Terlizzi et al. [36] reported the most abundant Italian isolates (seven isolates) in group III (group GRSPaV-SG1) and these isolates were obtained from *V*. *vinifera* cv. Biancale, Rossiola, Pagadebit and Canino. All these cultivars originated from Emilia Romagna, which is a region of Italy adjacent to Tuscany.

GRSPaV is one of the most scattered grapevine viruses in the world. It is spread through grafting, vegetative propagation [38] and perhaps via pollen and seed [39]. Despite the relatively high genetic diversity of the virus, there is an absence of geographical clustering of variants probably due to its widespread distribution through the viticulture industry and its transmission through the cultivated grapevines [40,3]. The detection of GRSPaV infection in wild grapevines suggests the presence of biological vectors (not yet identified) [6] involved in a short distance transmission in natural environments. This hypothesis regarding the virus epidemiology could explain the geographical clustering found for the Tuscan isolates from wild grapevines (they clustered in group GRSPaV-SG1 together with the seven Italian isolates collected by Terlizzi et al. [36] from cultivars originating from Emilia Romagna).

Since it is well known that recombination can affect phylogenetic reconstruction [33], the GRSPaV CP genes were initially analyzed for evidence of recombination events. The Recombination Detection Program v.3.34 (RDP4) was used to determine whether recombination events were present in the studied sequences. Heuristic recombination detection methods identify non-recombinant fragments in the alignment.

Analysis of the whole GRSPaV CP sequence confirmed the phylogenetic relationships of GRSPaV genotypes from Tuscan wild grapevines with isolates present in GenBank, and amino acid analysis supported the presence of motifs conserved in the GRSPaV coat proteins, such as the KRKR domain involved in the nuclear localisation of the viral capsid protein [41].

#### GLRaV-1 sequences

**Fig 3:**
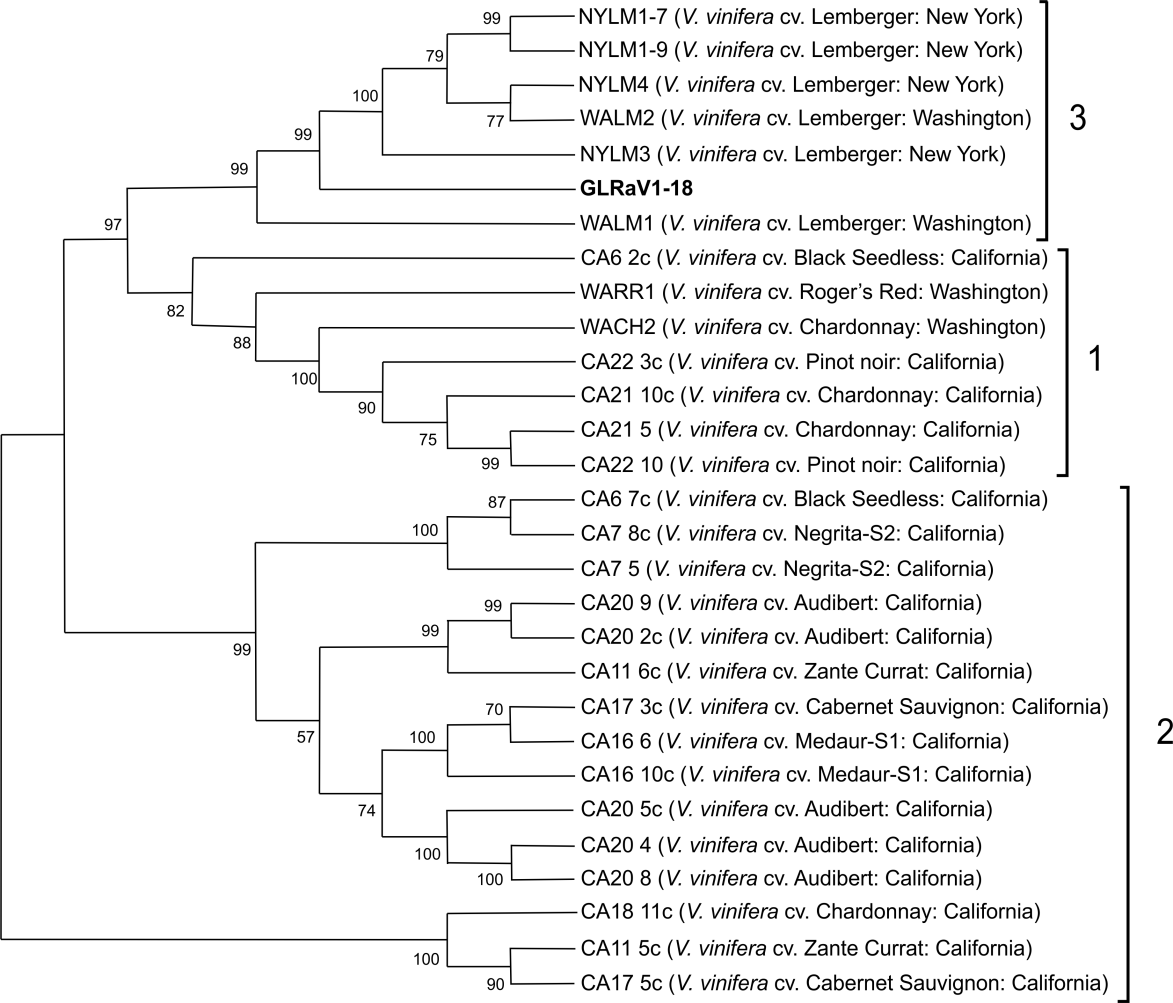
Phylogenetic tree showing the distribution of coat protein nucleotide sequences of Tuscan *Grapevine leafroll-associated virus 1* (GLRaV-1). The neighbour-joining tree shows the distribution of Tuscan GLRaV-1 coat protein nucleotide sequences compared to isolates from the GenBank. Geographical origin is given for each isolate (in brackets). Bootstrap values are from 1000 replicates.

**Fig 4:**
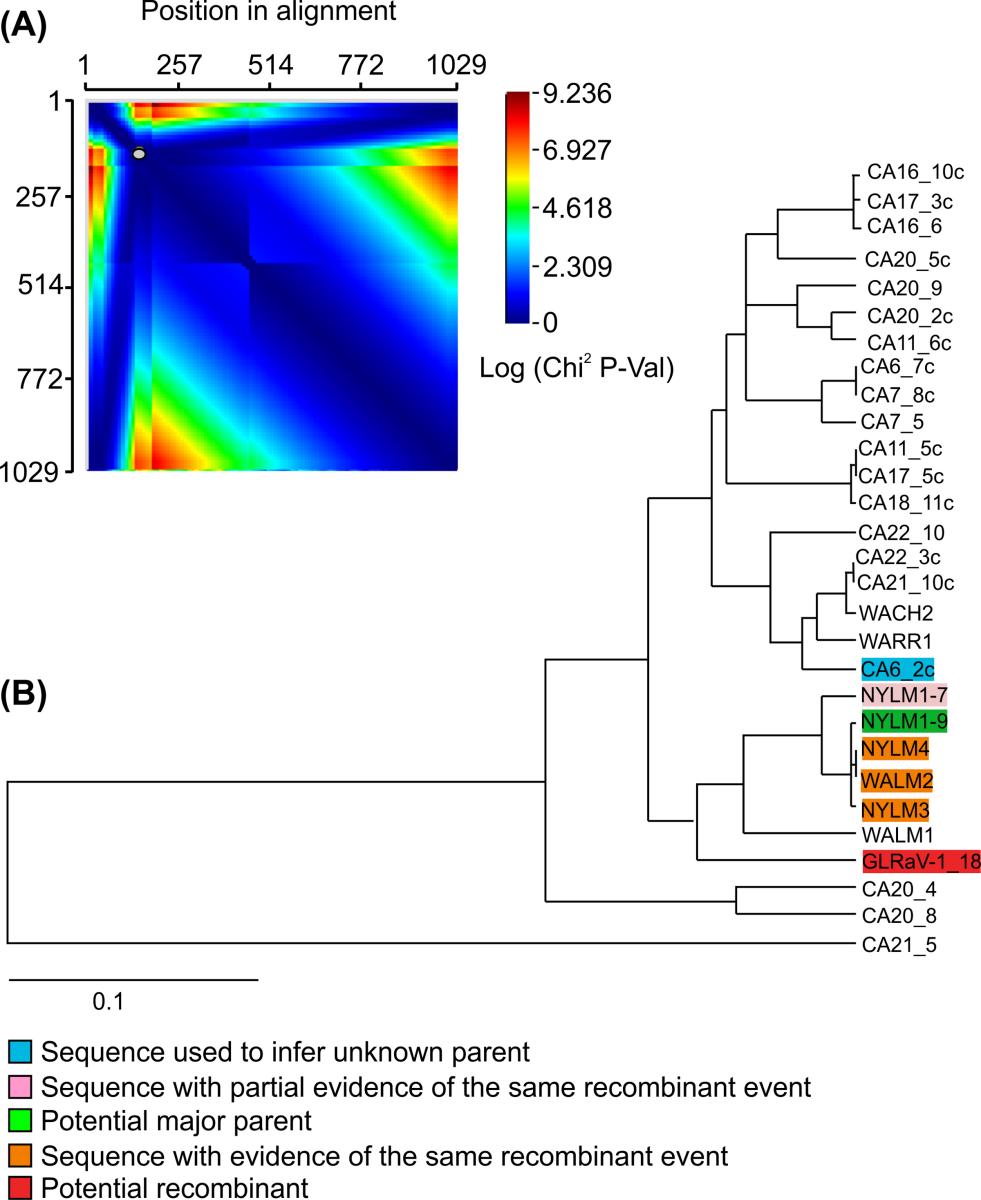
Recombination analysis by RDP4 of 29 coat protein nucleotide sequence alignments. (A) MaxChi breakpoint matrix. MaxChi matrices are useful for identifying the statistically optimal positions of breakpoint pairs. Colors represent chi-squared values for different pairs of breakpoints; dark red peaks indicate the most probable positions of breakpoint pairs. (B) Tree display providing information on parental sequences (and phylogenetically plausible alternative parents). The red highlighted sequence is the currently selected recombinant sequence; green and blue indicate reasonably close relatives of major and minor parents; orange and pink have similar (Orange) or somewhat similar but notably different (Pink) recombination signals to that observed in the sequence highlighted in red.

Alabi et al. [26] differentiated global GLRaV-1 isolates mainly into three variant groups based on CP gene sequence data: groups 1, 2 and 3. In our study, phylogenetic analysis of the CP gene sequence showed that GLRaV-1 isolate (S3 File) clustered with variants from group 3 (Fig 3). Group 3 consists of isolates originating from cv. Lemberger from Washington and New York. RDP4 analysis revealed a potential recombination event around position 55–129 (Fig 4A) and, in the analysed dataset, indicated the sequence NYLM1-9 (cv. Lemberger from New York, JF811855) as the potential major parent (90.4% similarity) and NYLM4, NYLM3 and WALM2 (respectively: cv. Lemberger from New York, JF811857, JF811856 and cv. Lemberger from Washington, JF811859) as those with evidence of the same recombination event (Fig 4B).

In many groups of viruses, genetic recombination generates much of the genetic diversity, however our results showed no evidence of clearly defined geographical structuring of GLRaV-1 isolates. This is possibly a consequence of their dissemination, primarily through infected grapevine cuttings. It further emphasizes the importance of the exchange and use of virus-free plant material in preventing the dissemination of this virus [26, 42].

The CP sequence generated in this study showed nucleotide sequence identities ranging from 66% to 87% with an overall ratio between the non-synonymous to the synonymous mutations (*dN/dS*) of < 1. These results showed a low number of non-silent mutations, indicating a purifying selection process. The amino acid sequence identities ranged from 70% to 91%, suggesting that the gene is under selection pressure probably to preserve encoded amino acid sequences and biological functions. Other works [43,44] have suggested a purifying selection on the genomic region of CP, probably due to functional constraints imposed on CP in many insect-transmitted viruses. Specific interactions between capsid proteins and cellular receptors in the insect vectors are necessary for successful transmission [43]. In fact, GLRaV-1 is transmitted by a species of mealybugs and scale insects [45].

#### GVA sequences

**Fig 5:**
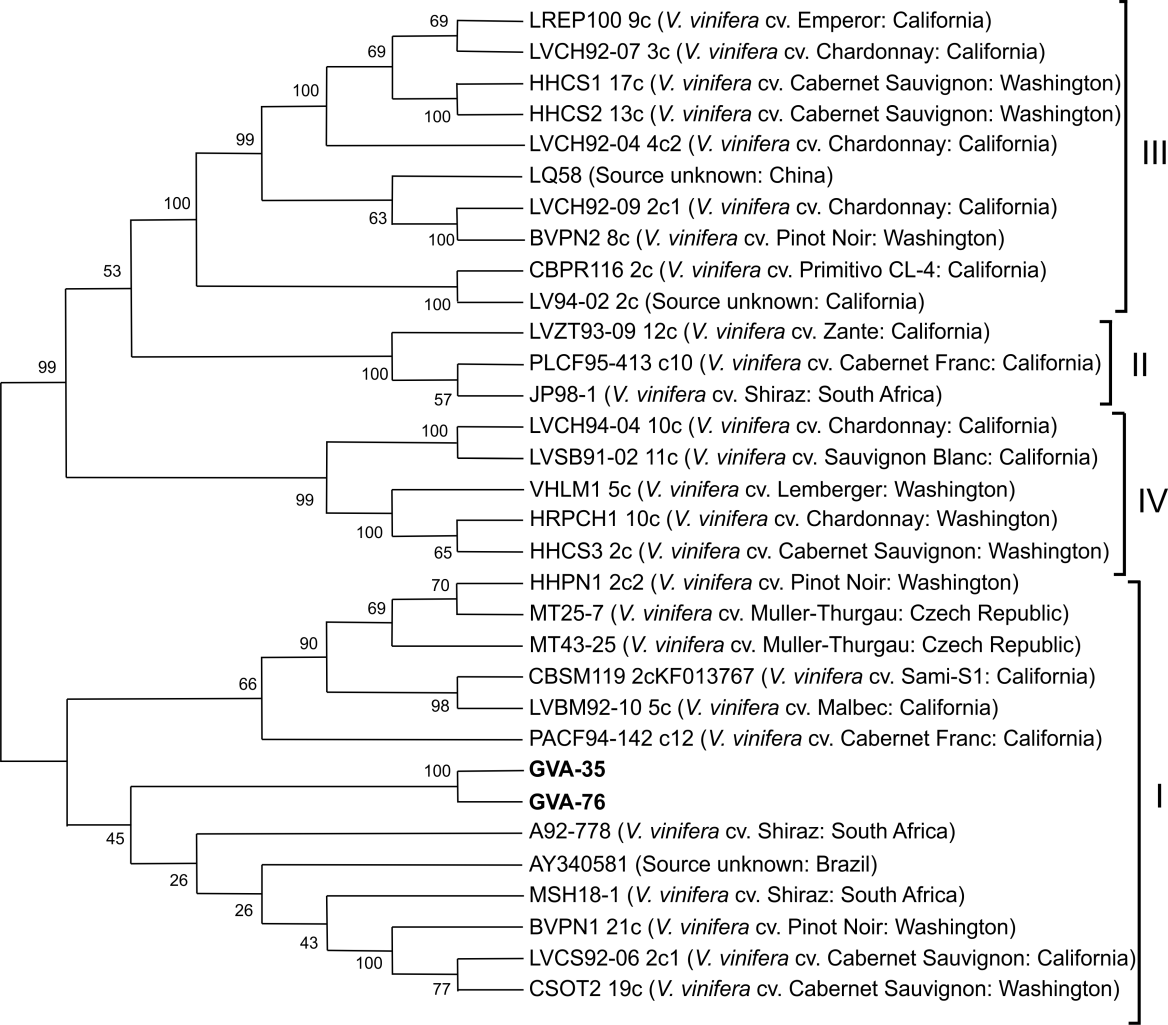
Phylogenetic tree showing the distribution of coat protein nucleotide sequences of Tuscan *Grapevine virus A* (GVA). Neighbour-joining tree shows the distribution of Tuscan GVA coat protein nucleotide sequences compared to isolates from the GenBank. The geographical origin is provided for each isolate (in brackets). Bootstrap values are from 1000 replicates.

GVA is distributed worldwide and is associated with Kober stem grooving and Shiraz diseases [46]. With a global phylogenetic analysis of CP sequences (from isolates collected from different wine grape cultivars) Alabi et al. [28] segregated virus isolates into four major clades: groups I, II, III and IV. GVA isolates from Tuscany wild grapevines (all the isolates identified from the 3 infected plants were sequenced and only the two representative isolates were included in the tree) (S3 File) showed a CP gene sequence identity of 98% with each other and clustered into group I (Fig 5), showing a nucleotide sequence identities ranging from 73.5% to 92%. According to the grouping proposed by Alabi et al. [28], group I was comprised of sequences from California, Washington, Czech Republic, Italy, South Africa, Brazil, and Israel. California, Washington, South Africa and Israel also clustered into other groups, while the Czech Republic, Italy and Brazil clustered only into group I, thus the global interpretation for GVA isolates from wine grape cultivars is that there is no geographical clustering. GVA isolates from Tuscany wild grapevines in this work clustered into the group including Italian samples. The overall ratio between the non-synonymous to the synonymous mutations (*dN/dS*) for the nucleotide sequences analyzed was < 1 with a low number of non-silent mutations, thus suggesting a purifying selection process. No evidence of recombination was found in the nucleotide sequence alignment using multiple methods within RDP4. Analysis of the amino acid CP sequences showed protein identity percentages ranging from 84% to 99%. These conserved regions are also in agreement with previous studies reporting GVA CP epitopes [47], indicating the existence of significant constraints to the modification of the amino acid composition of these structural proteins.

#### GLRaV-3 sequences

**Fig 6:**
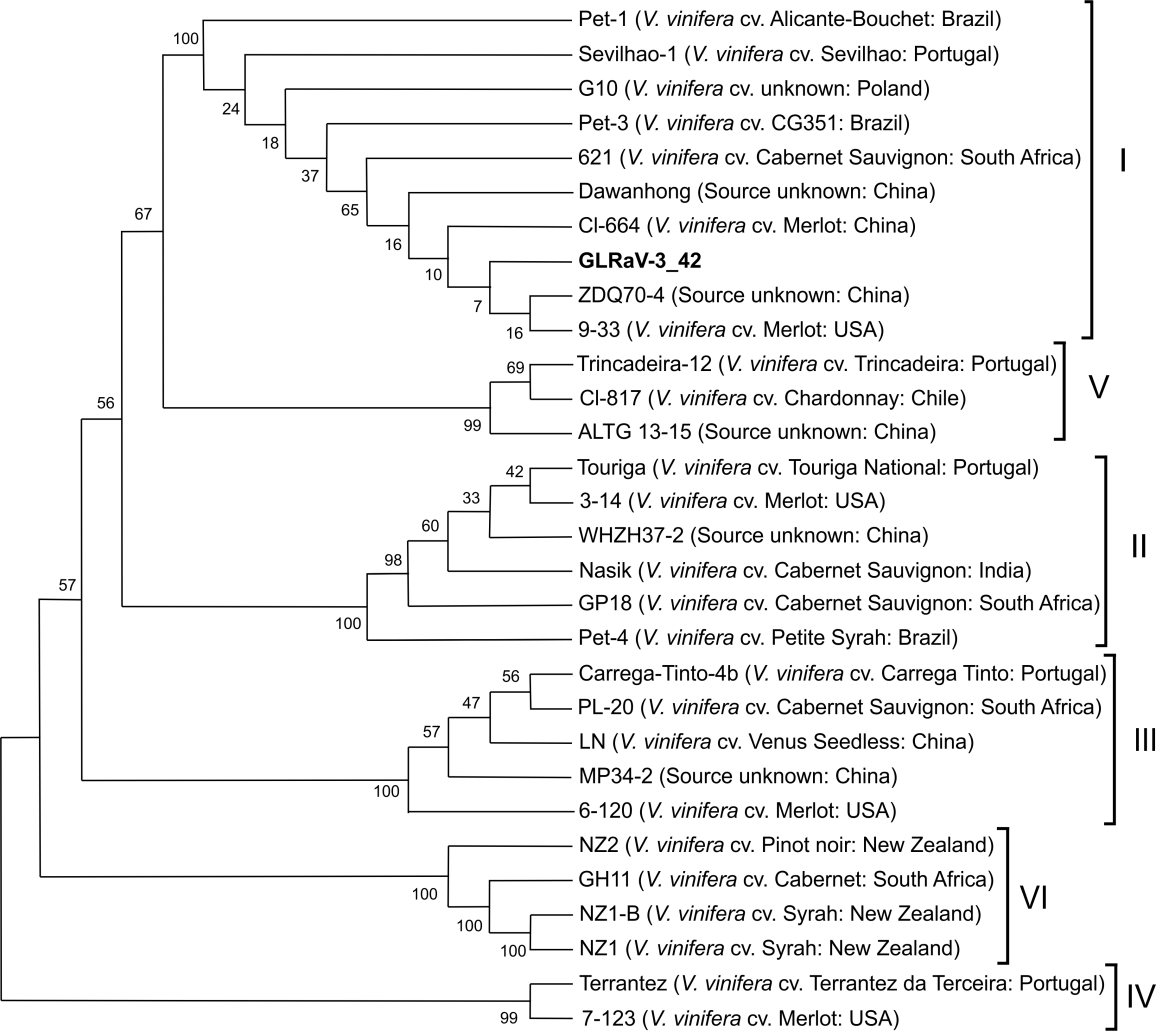
Phylogenetic tree showing the distribution of coat protein nucleotide sequences of Tuscan *Grapevine leafroll-associated virus 3* (GLRaV-3). Neighbour-joining tree shows the distribution of Tuscan GLRaV-3 coat protein nucleotide sequences compared to isolates from the GenBank. Geographical origin is provided for each isolate (in brackets). Bootstrap values are from 1000 replicates.

GLRaV-3 is regarded as the “main etiological agent” of Grapevine Leafroll Disease (GLD) which is one of the most important viral diseases affecting grapevines [35]. Six well-supported phylogenetic groups (I-VI) were detected in the analysis of full-length CP gene sequences deposited in GenBank [48]. GLRaV-3 isolate from Tuscany native *V*. *v*. *sylvestris* (S3 File) clustered in group I with nucleotide sequence identities ranging from 72.5% to 99% (Fig 6). Phylogenetic group I included genetic variants from China, South Africa, USA, Brazil, Poland and Portugal. Generally, all the genetic variants of GLRaV-3 have a worldwide distribution that could be attributed to the commercial trade of infected material, which demonstrates a lack of correlation between the viral variants and the geographical origin. The overall ratio between the non-synonymous to the synonymous mutations (*dN/dS*) for the nucleotide sequences analyzed was < 1 with a low number of non-silent mutations, thus highlighting a purifying selection process.

The results from RDP4 analysis indicated no evidence of recombination in the alignment of the compared isolates. Analysis of the amino acid CP sequences showed protein identity percentages ranging from 95.5% to 100%. The higher conservation of this protein is probably explained by its functional activity so that variation could affect the fitness of the virus. GLRaV-3 is phloem-limited and spread in the field through the transmission of several species of insects, and the specific interaction between capsid proteins and cellular receptors in the insect vectors is a key event [43].

## Conclusions

With regard to surveys carried out in Europe, the health status of *V*. *v*. *sylvestris* in Tuscany showed some differences. Leafroll disease was found to be approximately 14% (with two viruses), almost twice what was found in Austria (where only GLRaV-1 was observed), while in Portugal the disease was rare, and was undetected in Sicily. The prevalence of GLRaV-1 compared to GLRaV-3 was in contrast to findings in Tuscan cultivated grapevine, where GLRaV-3 was 7 times more frequent [9]. Considering other regulated viruses (following European or Italian regulations), GFLV has been found only in Sicily so far [1]. Although related to just one plant, this finding highlights the particular health status of Italian *V*. *v*. *sylvestris* compared to other European accessions. Conversely, this virus was not found in Tuscany, whereas GVA was quite common in wild Tuscan grapevines, thus indicating that rugose wood is a significant disease for *V*. *v*. *sylvestris* in this area. This finding is in line with previous surveys on *V*. *vinifera* in Tuscany, where GVA was frequently found [9]. Our findings showed that GRSPaV was the most widespread virus, in line with surveys in Portugal and Sicily where one mixed infection with a leafroll virus and no mixed infection was found, respectively [1]. Conversely, we observed three different mixed infections (also with a triple infection), which represent almost one-third of infected plants.

According to our phylogenetic analyses, GLRaV-1 and GLRaV-3 isolates from Tuscan wild grapevines clustered with isolates from cultivated grapevines without any correlation between distribution and geographical origin. On the other hand, GRSPaV and GVA isolates clustered in groups of genomic variants which appear also to be the most frequent for other analyzed Italian isolates found in *V*. *vinifera*.

It remains unclear as to how the wild *Vitis* species is infected with different variants of grapevine viruses. Two hypotheses are possible: one is that the ancestors of viruses co-evolved with *Vitis* spp. from the origin, diverging when the different *Vitis* species diverged; after which the viral variants were transferred into *V*. *vinifera* varieties through grafting. The second hypothesis is that the grapevine viruses may have existed in *V*. *vinifera* varieties and then could have been transmitted to wild grapevines by insect vectors or by human activities. Grapevines host many viruses, which is probably due to their extended cultivation and grafting [49].

The d*N*/d*S* ratio partially supports the first hypothesis. In fact, the ratio between the non-synonymous to the synonymous mutations for the CP genomic variants of GLRaV-1, GLRaV-3, GVA and GRSPaV isolates was always < 1, revealing the low number of non-silent mutations and highlighting a purifying selection of the viruses under study. These data could be related to a long-term form of adaptation and co-evolution between *V*. *v*. *sylvestris* and grapevine viruses. Once infected by viruses, vines remain infected throughout their lifetime and, generally, viruses are transmitted to vegetative progeny by insect vectors or humans, generating a host-pathogen system co-evolved in different *Vitis* species over time.

Once infected, wild grapevines may therefore act as potential virus reservoirs in regions where vector populations and wild grapevines are not usually monitored. In addition, the putative tolerance (no symptoms were detected in infected plants) observed in the wild grapevines, enforce the theory of the *V*. *v*. *sylvestris* as a reservoir of pathogens. Monitoring of wild grapevines is therefore essential both to detect viral infection, which is potentially dangerous for cultivated cultivars, and to adopt a conservation strategy. In fact, wild grapevines represent a valuable genetic resource for the breeding programs of cultivated grape.

Our findings on the health status of wild grapevines in Tuscany highlight the importance of regular phytosanitary surveys for both cultivated and wild grapevines, since the control and management of diseases depend on the accurate identification of their etiology.

## Supporting information

S1 File Absorbance (OD_405_) results of the ELISA tests for GLRaV-1, -3, GVA and GRSPaV. (XLSX)

S2 File Threshold cycle (Ct) values of the quantitative real-time PCR (qPCR) for GLRaV-1, -3, GVA and GRSPaV detection. (XLSX)

S3 File List of the coat protein (CP) nucleotide sequences of the Tuscan viral isolates used in the phylogenetic analysis. (PDF)

S1 Table List of GenBank coat protein sequences of the GRSPaV isolates used in this study. Name, cultivar, country and other details of GRSPaV isolates analysed in this study.
(PDF)

S2 Table List of GenBank coat protein sequences of the GLRaV-1 isolates used in this study. Name, cultivar, country and other details of GLRaV-1 isolates analysed in this study.
(PDF)

S3 Table List of GenBank coat protein sequences of the GVA isolates used in this study. Name, cultivar, country and other details of GVA isolates analysed in this study.
(PDF)

S4 Table List of GenBank coat protein sequences of the GLRaV-3 isolates used in this study. Name, cultivar, country and other details of GLRaV-3 isolates analysed in this study.
(PDF)
