# Supplementary material for: Phylogenetic analysis of viruses in Tuscan Vitis vinifera sylvestris (Gmeli) Hegi
Source: PLoS One. 2018 Jul 18;13(7):e0200875. doi: 10.1371/journal.pone.0200875 (PMC6051638; doi:10.1371/journal.pone.0200875)
Supplement: S3 File — (PDF) [file pone.0200875.s003.pdf]

S3 File

>GRSPaV-32

TGAAGGCTTTAGGGGTTAGCCAAAAAGTTGATGAAATTGATTACAGTTGTTGAAAGAG
TTAAATGGCAAGTCAAATTGGAAAGTTGCCTGGCGAGTCAAATGAAGCATATGAAGCC
AGACTTAAGGCTTTAGAGTTAGCAAGGGCTCAAAAGGCTCCGGAAGTTTCCAGCCAAC
CTCCCACACTTAGTGGCATTCTTGCCAAAAGGAAAAGAGTGATTGAGAATGCACTCTC
GAAGACAGTGGATATGCGTGAAGTTTTAAGGCATGAATCTGTTGTACTCTCCCCAAAT
GTGATGGATGAGGGAGCAATAGATGAGCTAATTCGTGCCTTTGGGGAGTCAGGCATAG
CTGAAAATGCGCAGTTCGATGTTGCAATAGACATTGCCCGCCACTGTTCTGATGTGGGA
AGTTCTCAGAGGTCAACCCTAATTGGTAAGAGCCCCTTCTGCGAGTTAAATCGGTCTGA
AATTGCTGGAATTATAAGGGAGGTAACCACACTGCGCAGATTTTGCATGTACTACGCA
AAAATCGTGTGGAACATTCATTTGGAGACGGGAATACCACCAGCTAATTGGGCCAAGA
AGGATTTAATGAGAATGAAAAGTTTGCAGCCTTTGACTTCTTCCTTGGAGTCACAGATG
AAAGCGCGCTTGAACCTAAGGGTGGTGTCAAGAGAGCTCCAACAAAGGCAGAGATGG
TCGCTAATATTGCCTCTTTTGAGGTCAAAGTGCTCAGACAGACTATGGCTGAAGGAAA
GCGGAGCTCCAATCTTGGAGAAATTAGTGGTGGAACGGCTGGGGCGCTTATTAACAAC
CCCTTTGCAAACGTTACGCATGAATGAGGTTGATAGTGGAAGCAATGCTCCTTCATCCA
AATAATCACCCGATTTCAAGGCTGGGT

>GRSPAV-50

GAAAAGAGTGATTGAGAATGCACTCTCAAAGGTAGACATGCGTGAAGTTTTAAGGCAT
GAATCTGTTGTGCTTTCCCCAAAAGTGATGGATGAGGGACCATCTATGAGCTGATTCGC
GCCTTTGGGGAGTCAGGCATAGCCGAGAATGCGCAAATTGATGTTGCAATAGACATTG
CCCGCCACTGTTCTGATGTAGGAAGTTCTCAATGGTCTACCCTATGTGGTAAAAGCCCC
TTCTGCGAGTTTAATCGGTCTGAAATTGCTGGAATTATAAGGGAGGTACCCACGCTGCG
TTCAACTTTGCTGTACTACGCAATAATTGTGTGGTACATCCATTTGGAGACGGGGAATA
CCACCGCCTAATTGGGCCATGAATGGATTTAATGAGAATGAAAAGGTTGATTCCTTTG
ACTTCTTCCTTGGAGTCACAGATGAGAGCGCGCTAGAACCTAAGGGTGGTGTCAAGAG
AGCTCCAACAAAAGCAGAGATGGTCGCTAATATTGCCTCTTTTGAAGTCAGAGTGCTC
ATACAGACTATGGCTGAAGGAAAGCGGAGATCCCATCTCGCAGAATTTAGAGGGGGG
AATCGGATGGGGCGCTCATCAAC

>GLRaV-1_18

ATGGCTAGCGTTATATCTCAAAATGATGATGACTATAACGTCGTCCGGGGAGGAAATA
TCGTTGTGCCGCGCACGCCTACTTTAAATGGTTTCGGTACATCGGCCTTCACTATACCT
GCAGGTGAGGCGACTGCGTACGTTTTGAAAACACAGTACTCGAAGCCAGAAGCTGGTA
CTCCCGAGGCAAATCACCCTGTGATTGGTGTTCTGCCAGACGAGTATGTGTTCGTGAAA
GGTCCGGGAGGTTATACTCTCCGTCCTTCTCGACCGTCTACGGGTCAGCGTTCGGGGGA

TTCTGAAGTGGCTAAGCGAGAAATGGGCGACCGGCCGCCATGGCGGCCGCGGGAATTCGATTTCTTTACCAACCCCGAGATGAATATTATCTTTGAGCCACCGAAGGATATGGAAGTTTCGGTTGTGGTGCCAACCGGACCCGGCTTGGTGACGCCGGCTGTGGCAACAGCTATATCTACGGAATTAAAAAATTTGTGTGCTGAAGTGATGGGTAATACTGATGAGAAAAGTCTCACAGATTTCTTCTTGGCAATGTTGCAATTAATGTTGACGTTTAGCACATCACCAGACACTGAGAGCAAAGAAGAGTACTTCGTGAATTTGTACAGTAACGGCGAGCGAAAATTAACTTACGAGAAGGTTAAGGGCGCCGTTGTCAAAGGAGCTGAAGGCTCGACGTTCGAAAATCCTATGCGTCAGTATGCCAGGTTGTTCTCAGCAACGGCTGTACATTTAATATTGAATGGAAAGTTAAGGCCGAACGAGAAAGTAGCTATGCAACACGGCGTACCTAAGAGGTTTCTTCCGTATACTTTTGATTTCTGCAGACCATCTTATTCTCAATTTAGCAATGACGCCATTAGAGCATGGCAGTTGGCGGCAGAATCGGCGTTCGGGCGGAAGAGTAATGTAACAAGCTCAGTACTAAGAAACACCAGTGAGCTCAAGGTGTAG

>GVA-35

GATACCCTAGTTATGCCAGAGGTGTATGAGACAATAAAGAAACTTGGGTTGAGGACAAATGGCACACTACGCCAAGAGGGTGGAGATAAGGGCGATAATAGAAGAGTTGGTGCTGGCGAAAGCCCAGCCAACTGAAGACGCTTCCGAGAGCGGCTACGACCGAAATATGTACCTGAATACTCTCTTCGGGTACATCGCCTTGGTCGGCACAAGCAAAAAGGCGGTCCATTATGGAGAGGTAGATATAGTAGGTCCTAAAGCTAGCAAAAAGACTGGGATAGACCCGAGAGGAAAACTGGTCGTATCTGAGCTAGTAGGCAGGATGCGCACACTGAGTGTGGCAGTCAGTGAGGGTCCTGTTAAAGGAGCAACTCTGAGGCAAATGTGCGAGCCATTCGCACAGAACGCCTATGACTTCCTCGTTCTGATGGCTGAGATGGGCACATATTCGCAGTTAGCAACTAAGATGACTAGGTCAGGCTTCAAGGAGCCACAGGTTATGTTCGACTTTGCGTCGGGCTTAGATCTGAAAGCACTGACATTGCAAGAAGCTACTGTGATACAGGCCATGCACTCCCGTCTCTTTCGTACGGAAGGCGCAAAGGGGGTATTCAACGCTCAGTCATCAGTCGGTGAGCAGGCTGTCGAAATATAGATGGATGACCCATCGTTTCTCTCGGGTAGGTCCACGTTTGCTAAGCGTAGGCGCGCTAGGCGCATGAATGTGTGGTAGTGTGGTGC

>GVA-76

ATGGCACACTACGCCAAGAGGGTGGAGATGAGGGCGATAATAGAAGAGTTGGTGCTGGCGAAAGCCCAGCCAACTGAAGACGCTTCAGAGAGCGGCTACGACCGAAATATGTACCTGAATACTCTCTTCGGGTACATCGCCTTGGTCGGCACAAGCAAAAAGGCGGTCCATTATGGAGAGGTAGATATAGTAGGTCCTAAAGCTAGCAAGAAGACAGGGATAGACCCGAGAGGAAAACTGGTCGTATCTGAACTAGTGGGCAGGATGCGCACACTGAGTGTGGCAGTCAGCGAGGGTCCTGTCAAAGGGGCAACTCTGAGGCAAATGTGCGAGCCATTCGCACAGAACGCCTATGACTTCCTCGTTCTGATGGCAGAGATGGGCACATATTCGCAGTTAGCAACCAAGATGACTAGGTCAGGCTTCAAGGAGCCACAGGTTATGTTCGACTTTGCGTCGGGCTTAGATCTGAAAGCACTGACATTGCAAGAAGCTACTGTGATACAGGCCATGCACTCCCGTCTCTTTCGTACGGAAGGCGCAAAGGGGGTATTCAACGCTCAGTCATCAGTCGGTGAGCAGGCTGTCGAAATATAGATGGATGACCCATCGTTTCTCTCGGGTAGGTCCACGTTTGCTAAGCGTAGGCGCGCTAGGCGCATGAATGTGTGTAAGTGTGGTGC

>GLRaV-3_42

TCCCCGAAAATAATTTGAGAGTTAGAGTAGGGGATGCGGCACGAGGAAAATTTAGTAA
GGCGAGTTTCTTAAAGTACGTTAAGGACGGACACAGGCGGAATTAACGGGAATCGCC
GTAGTGCCCGAAAAATACGTATTCGCCACAGCAGCTTTGGCTACAGCGGCGCAGGAGC
CACCTAGGCAGCCACCAGCGCAAGTGGTGGAACCACAGGAAACCGATATAGGGGTAG
TGCCGGAATCTGAGACTCTCACACCAAATAAGTTGGTTTTCGAGAAAGATCCAGACAA
GTTCTTGAAGACTATGGGCAAGGGAATAGCTTTGGACTTGACGGGAGTTACCCACAAA
CCGAAAGTTATTAACGAGCCAGGGAAAGTATCAGTAGAGGTGGCAATGAAGATTAAT
GCTGCATTGATGGAGCTGTGTAAGAAGGTTATGGGCGCCGATGACGCAGCAACTAAGA
CAAAATTCTTCTTGTACGTGATGCAGATTGCTTGCACGTTCTTTACATCGTCTTCGACGG
AGTTCAAAGAGTTTGACTACATAGAAACCGATGATGGAAAGAAGATATATGCGGTGTG
GGTATATGATTGCATTAAACAAGCTGCTGCTTCGACGGGTTATGAAAACCCGGTAAGG
CAGTATCTAGCGTACTTCACACCAACCTTTATCACGGCGACCCTGAATGGTAAACTAGT
GATGAACGAGAAGGTTATGGCACAGCATGGAGTACCACCGAAATTCTTTCCGTACACG
ATAGACTGCGTTCGTCCGACGTACGATCTGTTCAACAACGACGCAATACTAGCATGGA
ATTTAGCTAGACAGCAGGCGTTTAGAAACAAGACGGTAACGGCCGATAACACCTTACA
TAACGTCTTCCAACTATTGCAAAAGAAGTAGAA
