# Supplementary material for: Phylogenetic analysis of viruses in Tuscan Vitis vinifera sylvestris (Gmeli) Hegi
Source: PLoS One. 2018 Jul 18;13(7):e0200875. doi: 10.1371/journal.pone.0200875 (PMC6051638; doi:10.1371/journal.pone.0200875)
Supplement: S1 Table — Name, cultivar, country and other details of GRSPaV isolates analysed in this study. (PDF) [file pone.0200875.s004.pdf]

S1 Table

Isolate	Specie/Cultivar	Country	GenBank accession number	Reference
M5-G	*V. vinifera*/Padeiro	Portugal	AY927671	Nolasco et al., 2006
SL48-14	*V. vinifera*/Refosk	Slovenia	AY927688	Nolasco et al., 2006
Hiz3	*V. labruscana*/Pione	Japan	AB331432	Nakaune et al., 2008
GRSPaV-1	Unknown	USA	NC_001948	Meng et al., 1998
E105-G	*V. vinifera*	Portugal	AY927676	Nolasco et al., 2006
BOS3	*V. vinifera*/Cabernet F.	Italy	DQ364991	Terlizzi et al., 2010
B10-1	*V. vinifera*	Portugal	AY927680	Nolasco et al., 2006
Cf6	*V. vinifera*/Cabernet F.	Italy	DQ364994	Terlizzi et al., 2010
Hiz5	*V. labruscana*/Pione	Japan	AB331433	Nakaune et al., 2008
Hiz6	*V. labruscana*/Pione	Japan	AB331434	Nakaune et al., 2008
Ls3	*V. vinifera*/Lambrusco	Italy	DQ364983	Terlizzi et al., 2010
TRCV4	*V. vinifera*/Trebbiano	Italy	DQ364987	Terlizzi et al., 2010
Doberdò	*V. sylvestris*	Italy	DQ364988	Terlizzi et al., 2010
M31-35	*V. vinifera*/Loureiro	Portugal	AY927673	Nolasco et al., 2006
GRSPaV-SG1	*Vitis rupestris*/St George	USA	AY881626	Meng et al., 2005
SL38-20	*V. vinifera*/Refosk	Slovenia	AY927687	Nolasco et al., 2006
B11-2	*V. vinifera*	Portugal	AY927679	Nolasco et al., 2006
D10	*V. vinifera*/Touriga N.	Portugal	AY927672	Nolasco et al., 2006
BL1	*V. vinifera*/Biancale	Italy	DQ364979	Terlizzi et al., 2010
NE423PM	*V. vinifera*/Nebbiolo	Italy	DQ364989	Terlizzi et al., 2010
CNO9	*V. vinifera*/Canino	Italy	DQ364981	Terlizzi et al., 2010
BL11A	*V. vinifera*/Biancale	Italy	DQ364980	Terlizzi et al., 2010
RSS6	*V. vinifera*/Rossiola	Italy	DQ364985	Terlizzi et al., 2010
GRSPaV-BS	Hybrid/Bertille	USA	AY881627	Meng et al., 2005
PGD2	*V. vinifera*/Pagadebit	Italy	DQ364984	Terlizzi et al., 2010
NE423TP	*V. vinifera*/Nebbiolo	Italy	DQ364990	Terlizzi et al., 2010
Hiz7	*V. labruscana*/Pione	Japan	AB331435	Nakaune et al., 2008
OB1	*V. labruscana*/Pione	Japan	AB331418	Nakaune et al., 2008
CNO15	*V. vinifera*/Canino	Italy	DQ364982	Terlizzi et al., 2010
Cl29	*V. vinifera*/Alvarinho	Portugal	AY927678	Nolasco et al., 2006
A8.4	*V. vinifera*	Portugal	AY927670	Nolasco et al., 2006
Vs279-2	*V. sylvestris*	Portugal	AY927684	Nolasco et al., 2006
M37-12	*V. vinifera*/Borracal	Portugal	AY927675	Nolasco et al., 2006
TRCV1	*V. vinifera*/Trebbiano	Italy	DQ364986	Terlizzi et al., 2010
M31-37	*V. vinifera*/Loureiro	Portugal	AY927674	Nolasco et al., 2006
Vs284-21	*V. sylvestris*	Portugal	AY927685	Nolasco et al., 2006
Vs284-23	*V. sylvestris*	Portugal	AY927686	Nolasco et al., 2006
