# Supplementary material for: Phylogenetic analysis of viruses in Tuscan Vitis vinifera sylvestris (Gmeli) Hegi
Source: PLoS One. 2018 Jul 18;13(7):e0200875. doi: 10.1371/journal.pone.0200875 (PMC6051638; doi:10.1371/journal.pone.0200875)
Supplement: S2 Table — Name, cultivar, country and other details of GLRaV-1 isolates analysed in this study. (PDF) [file pone.0200875.s005.pdf]

S2 Table

Isolate	Specie/Cultivar	Country	GenBank accession number	Reference
NYLM1-7	*V. vinifera*/Lemberger	New York	JF811854	Alabi et al., 2011
NYLM1-9	*V. vinifera*/Lemberger	New York	JF811855	Alabi et al., 2011
NYLM4	*V. vinifera*/Lemberger	New York	JF811857	Alabi et al., 2011
NYLM2	*V. vinifera*/Lemberger	Washington	JF811826	Alabi et al., 2011
NYLM3	*V. vinifera*/Lemberger	New York	JF811832	Alabi et al., 2011
WALM1	*V. vinifera*/Lemberger	Washington	JF811858	Alabi et al., 2011
CA6 2c	*V. vinifera*/Black Seedless	California	JF811850	Alabi et al., 2011
WARR1	*V. vinifera*/Roger's Red	Washington	JF811861	Alabi et al., 2011
WACH2	*V. vinifera*/Chardonnnay	Washington	JF811860	Alabi et al., 2011
CA22 3c	*V. vinifera*/Pinot noir	California	JF811849	Alabi et al., 2011
CA21 10c	*V. vinifera*/Chardonnnay	California	JF811847	Alabi et al., 2011
CA21 5	*V. vinifera*/Chardonnnay	California	JF811846	Alabi et al., 2011
CA22 10	*V. vinifera*/Pinot noir	California	JF811848	Alabi et al., 2011
CA6 7c	*V. vinifera*/Black Seedless	California	JF811851	Alabi et al., 2011
CA7 8c	*V. vinifera*/Negrita-S2	California	JF811853	Alabi et al., 2011
CA7 5	*V. vinifera*/Negrita-S2	California	JF811852	Alabi et al., 2011
CA20 9	*V. vinifera*/Audibert	California	JF811843	Alabi et al., 2011
CA20 2c	*V. vinifera*/Audibert	California	JF811844	Alabi et al., 2011
CA11 6c	*V. vinifera*/Zante Currat	California	JF811834	Alabi et al., 2011
CA17 3c	*V. vinifera*/Cabernet Sauvignon	California	JF811838	Alabi et al., 2011
CA16 6	*V. vinifera*/Medaur - S1	California	JF811836	Alabi et al., 2011
CA16 10C	*V. vinifera*/Medaur - S1	California	JF811837	Alabi et al., 2011
CA20 5c	*V. vinifera*/Audibert	California	JF811845	Alabi et al., 2011
CA20 4	*V. vinifera*/Audibert	California	JF811841	Alabi et al., 2011
CA20 8	*V. vinifera*/Audibert	California	JF811842	Alabi et al., 2011
CA18 11c	*V. vinifera*/Chardonnnay	California	JF811840	Alabi et al., 2011
CA11 5c	*V. vinifera*/Zante Currat	California	JF811833	Alabi et al., 2011
CA17 5c	*V. vinifera*/Cabernet Sauvignon	California	JF811839	Alabi et al., 2011
