# Supplementary material for: Phylogenetic analysis of viruses in Tuscan Vitis vinifera sylvestris (Gmeli) Hegi
Source: PLoS One. 2018 Jul 18;13(7):e0200875. doi: 10.1371/journal.pone.0200875 (PMC6051638; doi:10.1371/journal.pone.0200875)
Supplement: S3 Table — Name, cultivar, country and other details of GVA isolates analysed in this study. (PDF) [file pone.0200875.s006.pdf]

S3 Table

Isolate	Specie/Cultivar	Country	GenBank accession number	Reference
LREP100 9c	*V. vinifera*/Emperor	California	KF013810	Alabi et al., 2014
LVCH92-07 3c	*V. vinifera*/Chardonnay	California	KF013819	Alabi et al., 2014
HHCS1 17c	*V. vinifera*/Cabernet Sauvignon	Washington	KF013786	Alabi et al., 2014
HHCS2 13c	*V. vinifera*/Cabernet Sauvignon	Washington	KF013788	Alabi et al., 2014
LVCH94-04 4c2	*V. vinifera*/Chardonnay	California	KF013816	Alabi et al., 2014
LQ58	Unknown	China	DQ911145	Alabi et al., 2014
LVCH92-09 2c1	*V. vinifera*/Chardonnay	California	KF013824	Alabi et al., 2014
BVPN2 8c	*V. vinifera*/ Pinot noir	Washington	KF013758	Alabi et al., 2014
CBPR116 2c	*V. vinifera*/ Primitivo CL-4	California	KF013763	Alabi et al., 2014
LV94-02 2c	Unknown	California	KF013812	Alabi et al., 2014
LVZT93-09 12c	*V. vinifera*/Zante	California	KF013842	Alabi et al., 2014
PLCF95-413 c10	*V. vinifera*/Cabernet Franc	California	KF013854	Alabi et al., 2014
JP98-1	*V. vinifera*/Shiraz	South Africa	AF441235	Goszczynski et al., 2003
LVCH94-04 10c	*V. vinifera*/Chardonnay	California	KF013828	Alabi et al., 2014
LVSB91-02 11c	*V. vinifera*/ Sauvignon Blanc	California	KF031841	Alabi et al., 2014
VHLM1 5c	*V. vinifera*/Lemberger	Washington	KF013806	Alabi et al., 2014
HRPCH1 10c	*V. vinifera*/Chardonnay	Washington	KF013797	Alabi et al., 2014
HHCS3 2c	*V. vinifera*/Cabernet Sauvignon	Washington	KF013790	Alabi et al., 2014
HHPN1 2c2	*V. vinifera*/Pinot noir	Washington	KF013793	Alabi et al., 2014
MT25-7	*V. vinifera*/Muller-Thurgau	Czech Republic	EU008561	Kominek et al., 2008
MT43-25	*V. vinifera*/Muller-Thurgau	Czech Republic	EU008560	Kominek et al., 2008
CBSM119 2c	*V. vinifera*/Sami-S1	California	KF013767	Alabi et al., 2014
LVMB92-10 5c	*V. vinifera*/Malbec	California	KF013836	Alabi et al., 2014
PACF94-142 c12	*V. vinifera*/Cabernet Franc	California	KF013849	Alabi et al., 2014
A92-778	*V. vinifera*/Shiraz	South Africa	AF441234	Goszczynski et al., 2003
AY340581	Unknown	Brazil	AY340581	Alabi et al., 2014
MSH18-1	*V. vinifera*/Shiraz	South Africa	DQ855085	Goszczynski et al., 2007
BVPN1 21c	*V. vinifera*/Pinot noir	Washington	KF013755	Alabi et al., 2014
LVCS92-06 2c1	*V. vinifera*/Cabernet Sauvignon	California	KF013830	Alabi et al., 2014
CSOT2 19c	*V. vinifera*/Cabernet Sauvignon	Washington	KF013775	Alabi et al., 2014
