# Supplementary material for: Phylogenetic analysis of viruses in Tuscan Vitis vinifera sylvestris (Gmeli) Hegi
Source: PLoS One. 2018 Jul 18;13(7):e0200875. doi: 10.1371/journal.pone.0200875 (PMC6051638; doi:10.1371/journal.pone.0200875)
Supplement: S4 Table — Name, cultivar, country and other details of GLRaV-3 isolates analysed in this study. (PDF) [file pone.0200875.s007.pdf]

S4 Table

Isolate	Specie/Cultivar	Country	GenBank accession number	Reference
Pet-1	*V. vinifera*/Alicante-Bouchet	Brazil	DQ680141	Maree et al., 2013
Sevilhao-1	*V. vinifera*/Sevilhao	Portugal	HQ401017	Gouveia et al., 2011
G10	*V. vinifera*/unknown	Poland	JN546586	Maree et al., 2013
Pet-3	*V. vinifera*/CG351	Brazil	DQ062152	Maree et al., 2013
621	*V. vinifera*/Cabernet Sauvignon	South Africa	GQ352631	Jooste et al., 2010
Dawanhong	unknown	China	DQ119574	Maree et al., 2013
Cl-664	*V. vinifera*/Merlot	China	EU344895	Engel et al., 2008
ZDQ70-4	unknown	China	JX088138	Maree et al., 2013
9-33	*V. vinifera*/Merlot	USA	HQ130329	Wang et al., 2011
Trincadeira-12	*V. vinifera*/Trincadeira	Portugal	HQ401016	Gouveia et al., 2011
Cl-817	*V. vinifera*/Chardonnay	Chile	EU344894	Engel et al., 2008
ALTG 13-15	unknown	China	JX088182	Maree et al., 2013
Touriga	*V. vinifera*/Touriga National	Portugal	HQ401019	Gouveia et al., 2011
3-14	*V. vinifera*/Merlot	USA	HQ130289	Wang et al., 2011
WHZH37-2	unknown	China	JX088172	Maree et al., 2013
Nasik	*V. vinifera*/Cabernet Sauvignon	India	JN616386	Kumar et al., 2012
GP18	*V. vinifera*/Cabernet Sauvignon	South Africa	EU259806	Maree et al., 2008
Pet-4	*V. vinifera*/Petite Syrah	Brazil	AY753208	Maree et al., 2013
Carrega-Tinto-4b	*V. vinifera*/Carrega Tinto	Portugal	HQ401018	Gouveia et al., 2011
PL-20	*V. vinifera*/Cabernet Sauvignon	South Africa	GQ352633	Jooste et al., 2010
LN	*V. vinifera*/Venus Seedless	China	JQ423939	Fei et al., 2013
MP34-2	unknown	China	JX088234	Maree et al., 2013
6-120	*V. vinifera*/Merlot	USA	HQ130305	Wang et al., 2011
NZ2	*V. vinifera*/Pinot Noir	New Zealand	JX220899	Chooi et al., 2013
GH11	*V. vinifera*/Cabernet	South Africa	JQ655295	Bester et al., 2012
NZ1-B	*V. vinifera*/Syrah	New Zealand	JX220900	Chooi et al., 2013
NZ1	*V. vinifera*/Syrah	New Zealand	EF508151	Maree et al., 2013
Terrantez	*V. vinifera*/Terrantez de Terceira	Portugal	HQ401015	Gouveia et al., 2011
7-123	*V. vinifera*/Merlot	USA	HQ130311	Wang et al., 2011
